# Analytical Blind Beamforming for a Multi-Antenna UAV Base-Station Receiver in Millimeter-Wave Bands

**DOI:** 10.3390/s21196561

**Published:** 2021-09-30

**Authors:** Pingchuan Liu, Kuangang Fan, Yuhang Chen

**Affiliations:** 1 School of Mechanical and Electrical Engineering, Jiangxi University of Science and Technology, Hongqi Street No. 86, Ganzhou 341000, China; wsnpingchuan@163.com; 2 Magnetic Suspension Technology Key Laboratory of Jiangxi Province, Jiangxi University of Science and Technology, Hongqi Street No. 86, Ganzhou 341000, China; chenyuhang@jxust.edu.cn; 3 School of Electrical Engineering and Automation, Jiangxi University of Science and Technology, Hongqi Street No. 86, Ganzhou 341000, China

**Keywords:** UAV base station, MIMO, millimeter-wave band, blind beamforming, signal recovery

## Abstract

Over the last decade, unmanned aerial vehicles (UAVs) with antenna arrays have usually been employed for the enhancement of wireless communication in millimeter-wave bands. They are commonly used as aerial base stations and relay platforms in order to serve multiple users. Many beamforming methods for improving communication quality based on channel estimation have been proposed. However, these methods can be resource-intensive due to the complexity of channel estimation in practice. Thus, in this paper, we formulate an MIMO blind beamforming problem at the receivers for UAV-assisted communications in which channel estimation is omitted in order to save communication resources. We introduce one analytical method, which is called the analytical constant modulus algorithm (ACMA), in order to perform blind beamforming at the UAV base station; this relies only on data received by the antenna. The feature of the constant modulus (CM) is employed to restrict the target user signals. Algebraic operations, such as singular value decomposition (SVD), are applied to separate the user signal space from other interferences. The number of users in the region served by the UAV can be detected by exploring information in the measured data. We seek solutions that are expressible as one Kronecker product structure in the signal space; then, the beamformers that correspond to each user can be successfully estimated. The simulation results show that, by using this analytically derived blind method, the system can achieve good signal recovery accuracy, a reasonable system sum rate, and acceptable complexity.

## 1. Introduction

Over the last few decades, wider bandwidths and more robust data transformations have always been the trends of the development of wireless communication. As one new and promising technology, millimeter-wave (mmWave) techniques have provided the possibility of applying upcoming 5G technologies, such as massive multiple-input–multiple-output (MIMO) technologies, to wireless systems [1,2,3,4].

One of the highlighted application scenarios of mmWave communication is in unmanned aerial vehicle (UAVs); such scenarios are presented with a base station, communication relay platform, or other communication enhancement equipment [1,2,3,4,5,6,7,8,9,10,11,12]. For instance, Z. Xiao et al. declared the advantages and challenges of mmWave UAV base stations (UAV-BSs) in cellular networks and explored different ways of counteracting signal blockage [2]. J. Du et al. studied UAV-BSs using multi-user massive MIMO hybrid beamforming and took an energy-saving strategy into consideration due to practical constraints, such as the size and weight of UAVs [3]. In addition to energy and power constraints, the mobility and positions of ground users are also crucial factors for UAV-assisted communication systems. In Reference [4], by exploiting the mobility and position information of a UAV system and ground users, W. Miao et al. proposed a lightweight beamforming algorithm in order to enhance the transmission performance of 5G UAV broadcasting system. In Reference [5], by using a UAV as a relay platform, F. Jiang and A. L. Swindlehurst studied the collection of messages from co-channel users on the ground and derived an algorithm for adjusting the UAV heading to maximize the approximate ergodic sum rate of the uplink channel. In some application scenarios, single-antenna UAVs are regarded as connection points or moving antennas for the enhancement of data transmission. For example, J. Ouyang et al. used a single-antenna UAV as a data transmission relay to connect access points and base stations [6]. M. Mozaffari et al. designed a novel framework for deploying a single-antenna UAV as an antenna array in order to minimize the wireless transmission time [8]. Some other recent research using single-antenna UAV-BSs has considered other scenarios [13,14,15].

In the research mentioned above and in other related research, usually, linear or planar antenna arrays are employed on the UAV-BSs. It is clear that adopting MIMO systems and mmWave communication in UAV-BSs has some convincing advantages. One aspect is that mmWave systems have rich spectral resources and flexible beamforming, which can meet the great demand of the data transfer rate. On the other hand, at particular positions, multi-antenna UAV-BSs can achieve high beamforming gain towards different line-of-sight (LoS) users by using mmWave communication, thus resulting in a more satisfying system throughput and communication efficiency [16,17,18,19,20,21,22]. However, due to some practical system constraints, such as multipath effects, blockage, and co-channel interference from different users, a UAV-BS system will sometimes have the issues of a low signal-to-noise ratio (SNR) in data acquisition and a low communication efficiency when serving multiple users.

To overcome these shortcomings and better utilize the advantages of mmWave, various beamforming techniques have been intensively investigated in UAV communication systems [16,17,18,19,20,21,23,24,25,26]. For example, Q. Song et al. studied the joint design of beamforming and power allocation to maximize the instantaneous data rate based on an efficient sub-optimal solution based on the block-coordinate descent method [23]. Some location-based beamforming algorithms were designed to improve the secrecy outage probability performance, maximize the network throughput, and provide flexible coverage [24,25,26]. In Reference [16], L. Liu et al. proposed a cooperative interference cancellation strategy motivated by the Xhaul structure to mitigate the uplink multi-beam UAV communication interference. In general communication systems, LoS transmission can efficiently improve the throughput of the UAV-BS. However, in urban areas, LoS links are prone to severe deterioration due to the complex propagation environments. In References [17,18], reconfigurable intelligent reflecting surfaces were used to reflect the received signals in order to enhance the data transmission quality, and passive beamforming was performed to better maximize the average achievable rate and received power. Combined with machine learning and a mean field game (MFG) control scheme, L. Li et al. proposed a joint beamforming and beam-steering method in order to build a reliable and steady connection between a UAV-BS and ground users in Reference [19]. Z. Xiao et al. utilized the artificial bee colony (ABC) algorithm to find the near-optimal beamforming vector for ground users when deploying a multi-antenna UAV-BS [20]. In Reference [21], W. Zhang et al. designed a beam-training code book for moving ground users, which can be regarded as a prior work of UAV-BS beamforming.

In this research and with the most recent beamforming techniques in UAV-BS communication systems, obtaining channel state information (CSI) is usually regarded as a crucial part of prior work. The effectiveness of channel estimation/identification may greatly affect the performance of most existing methods. In practical scenarios, especially in urban areas, specific CSI is difficult to acquire due to the complex propagation environment and multi-user interference. Another factor that greatly affects the channel estimation is the mobility of transmitters and receivers. The transmitting distance can also affect the performance of channel estimation. Accurate time-varying MIMO channel estimation can be very difficult to realize [4,7,20,27]. Moreover, the positions and mobility of ground users and UAV-BSs further increase the difficulty of channel estimation [28]. This puts extra pressure on the timeliness of a system, which causes bad beamforming performance. For this reason, we would like to formulate a blind beamforming problem for a UAV-BS that does not depend on channel estimation or CSI.

Recently, some works explored beamforming methods that needed less specific CSI. For example, X. Li et al. derived a beamforming method for different users under the assumption of only statistical CSI, i.e., the LoS component and Rician K-factor [29]. L. Du et al. proposed a robust pre-coder using imperfect CSI [30]. A beamforming scheme considering limited CSI was proposed with the aid of full-diversity rotation matrices [31]. However, these beamforming methods still partially rely on channel estimation, which can be time-consuming in complicated propagation environments. To tackle the thorny problem mentioned above that is caused by complicated channel estimation, in this paper, we will employ a method called the analytical constant modulus algorithm (ACMA) in order to perform blind beamforming in UAV-BS communication systems. Differently from methods that require detailed CSI, this blind method needs only the antenna output signals (the received signals) and some of their statistical information. In other words, it saves the computational costs caused by complex channel estimation. Another advantage of the blind beamforming method is that no training sequence is needed in the data transmission process, which saves the bandwidth and UAV’s battery life. The task of blind beamforming is to calculate proper weight vectors for each ground user without detailed signal and channel information [32,33,34].

Compared with a traditional blind beamforming method called the constant modulus algorithm (CMA), the ACMA scheme can overcome the CMA’s disadvantages of a lengthy iteration process and irregular convergence to local minima [32]. The analytical scheme can successfully derive a proper beamformer for the UAV-BS system without CSI. It is, in essence, a space separation method, which needs no lengthy iterations and achieves near-optimal accuracy. Using algebraic operations, such as singular value decomposition and diagonalization, we can successfully separate the user signal space (signals originally transmitted by ground users) from interference space. By properly investigating the properties of antenna outputs, this method can achieve near-optimal calculations of the weight vectors. These well-calculated weight vectors will finally formulate blind beamformers in order to assign the power of each antenna towards the corresponding ground users. At the same time, co-channel interference among different users will be efficiently compensated, and the desired signals can be successfully separated from other signals. We will evaluate this method’s performance by applying it to a UAV-BS communication system in later sections. Different propagation settings are taken into consideration. The simulation results show that, through this analytical blind method, UAV-BS beamforming in an MIMO situation can provide a reasonable system sum rate while, at the same time, guaranteeing the signal reconstruction accuracy of each signal. Its complexity is comparable to that of the CMA and other classic methods under proper settings.

The main contributions of this paper are listed below:

- In this paper, we solve the UAV-BS beamforming problem in a blind way, which means that channel estimation is not necessary in our work. We only explore information from antenna outputs at the UAV-BS. This will save computation resources for the whole communication system.
- The number of UAV-serving users (ground users) can be detected in the scheme for sufficient snapshots and a reasonable signal–noise ratio. Algebraic operations can successfully separate the user signal space from other interference spaces, thus causing the beamformers to concentrate on user signals instead of other interference signals.
- With sufficient snapshots, this analytical blind method is robust for different channel settings. It can suppress distortions caused by both interference and additive noise. A near-optimal beamformer can be rapidly derived even when the number of snapshots is not very large. The simulation results show that the derived beamformer can achieve good MSE performance, a reasonable system sum rate, and acceptable complexity. One potential UAV-BS application scenario can be a situation in which some specific users/target signals need higher-quality communications

The rest of this paper is organized as follows: Section 2 introduces the basic models for the UAV-BS communication system. In Section 3, we formulate a blind beamforming problem. Section 4 demonstrates the way in which we solve the formulated problem. In Section 5, we evaluate the performance of our introduced method under different settings.

*Notations:* We denote scalars, vectors, and matrices by using lowercase *a*, bold lowercase a, and bold uppercase letters A, respectively. We further denote the transpose, element-wise complex conjugation, and conjugate transpose of a matrix A by $A^{{}^{T}}$, $A^{*}$, and $A^{H}$, respectively. 0 denotes an all-zero-element vector or matrix of appropriate size. Moreover, ⊗ denotes a Kronecker product, whereas pinv (A), SVD (A), and rowspan(A) represent a pseudo-inverse, singular value decomposition, and row span of matrix A. Finally, ${\left(A\right)}_{i}$ denotes the *i*-th row, $A^{j}$ the *j*-th column, and $A_{ij}$ the element in row *i* and column *j* of matrix A, respectively.

## 2. Basic Model

### 2.1. System Model

**Figure 1:**
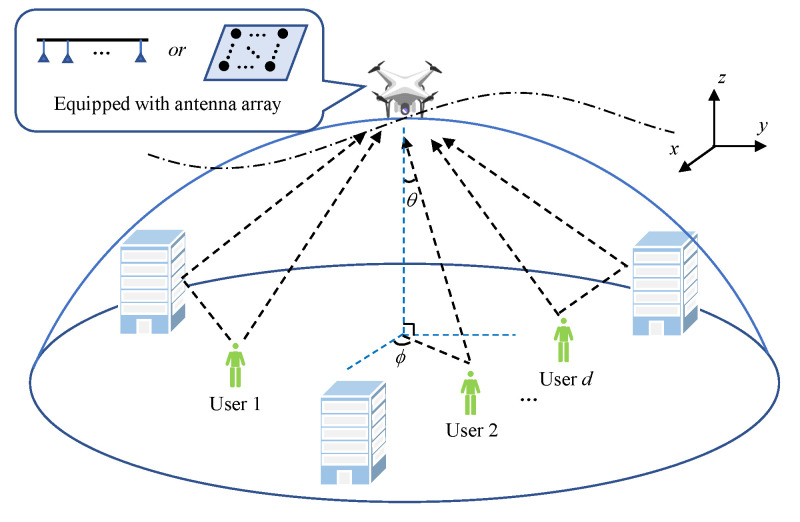
Application scenario of a UAV-BS serving multiple users.

In this paper, we consider an MIMO uplink scenario in which a multi-antenna UAV-BS serves ground users in mmWave bands, as shown in Figure 1. In this scenario, the UAV-BS is usually equipped with a half-wavelength-spaced uniform linear array (ULA) or uniform rectangular array (URA), while each ground user is equipped with a single antenna. The ground users are distributed among distinct locations, transmitting signals of the same frequency. Due to the existence of scattering and a reflective environment, a multi-path effect is also considered in this scenario. We first introduce the well-known model for describing this signal transmission process:

(1) $$\begin{array}{c} X=\mathrm{HS}+N, \end{array}$$

where $X\in C^{m\times n}$ is the output of the *m*-element receiver on the UAV-BS over *n* symbol snapshots, $H\in C^{m\times d}$ is the channel matrix between users and the UAV-BS, and $S\in C^{d\times n}$ is the source signal matrix transmitted by ground users. Each $s_{i}^{T}={\left(S\right)}_{i}\in C^{n},i\in \left\{1,\ldots ,d\right\}$ corresponds to *d* ground users. $N\in C^{m\times n}$ denotes the channel noise matrix.

To fight against the impacts of the channels on the signals, beamforming is usually applied at the transmitters or receivers (sometimes both).

In this paper, we focus on beamforming (we can also call it beam-combining) at the UAV-BS receivers, which can be modeled as

(2) $$\begin{array}{c} \hat{S}=\mathrm{WX}. \end{array}$$

The beamforming matrix $W\in C^{d\times m}$ is applied to compensate for the channels’ impacts, especially for interference cancellation, power allocation, further signal recovery processes, and so on. The rows of W, which are denoted as $w_{i}^{T}={\left(W\right)}_{i},\;i\in \left\{1,\ldots ,d\right\}$, correspond to the beamforming vectors for each ground user.

### 2.2. Channel Model

We then establish a 3D rectangular coordinate system to better illustrate the positions of the UAV-BS and ground users. The UAV-BS is located at $\left(x,y,h_{uav}\right)$, where $h_{uav}$ represents the UAV’s flying altitude. The position of ground user *i* is $\left(x_{i},y_{i},0\right)$.

The channel matrix H consists of channel response vectors between users and the UAV-BS. In the classic 2D situation, due to the existence of multi-path components (MPCs), each channel vector $h_{i}$ can be expressed as

(3) $$\begin{array}{c} h_{i}=\sum_{l=1}^{L_{i}}\kappa_{i,l}a\left(m,\theta_{i,l}\right), \end{array}$$

where $\kappa_{i,l}$ represents the channel gain coefficient corresponding to the *l*-th MPC of user *i*, $a(m,\theta_{i,l})$ is the steering vector, *m* is the number of antenna elements, $L_{i}$ is the number of existing MPCs, and $\theta_{i,l}$ is the elevation angle of arrival (AoA) of the l−th MPC. Due to the spatial sparsity of the angles of arrival in the mmWave channel, different MPCs will have distinct AoAs. The steering vector can then be derived as

(4) $$\begin{array}{c} a\left(m,\theta_{i,l}\right)={\left[\begin{array}{c} 1,e^{j\pi \sin\theta_{i,l}},e^{j\pi 2\sin\theta_{i,l}}\ldots ,e^{j\pi \left(m-1\right)\sin\theta_{i,l}} \end{array}\right]}^{T}. \end{array}$$

Equation (4) illustrates the 2D situation, though when applying 3D beamforming by using a uniform rectangular array (URA), the steering vector will be slightly different. For a half-spaced URA, the steering vector can be derived as

(5) $$\begin{array}{c} a\left(m_{x},m_{y},\theta_{i,l},\phi_{i,l}\right)=a_{x}\left(m_{x},\theta_{i,l},\phi_{i,l}\right)⊗a_{y}\left(m_{y},\theta_{i,l},\phi_{i,l}\right), \end{array}$$

where $m_{x}$ and $m_{y}$ are the URA dimensions along the x−axis and y−axis directions, and the total array elements are $m=m_{x}*m_{y}$. $\phi_{i,l}$ is the azimuth angle of the l−th MPC. The geometry denoted with $a_{x}$ and $a_{y}$ can further be described as

(6a) $$\begin{array}{cc} \; & a_{x}\left(m_{x},\theta_{i,l},\phi_{i,l}\right)={\left[\begin{array}{c} 1,e^{j\pi \sin\theta_{i,l}\cos\phi_{i,l}},\ldots ,e^{j\pi \left(m_{x}-1\right)\sin\theta_{i,l}\cos\phi_{i,l}} \end{array}\right]}^{T}, \end{array}$$

(6b) $$\begin{array}{cc} \; & a_{y}\left(m_{y},\theta_{i,l},\phi_{i,l}\right)={\left[\begin{array}{c} 1,e^{j\pi \sin\theta_{i,l}\sin\phi_{i,l}},\ldots ,e^{j\pi \left(m_{y}-1\right)\sin\theta_{i,l}\sin\phi_{i,l}} \end{array}\right]}^{T}. \end{array}$$

Generally, the MPCs between the UAV-BS and ground users are composed of the LoS part and non-LoS (NLoS) part under the condition of no blockage. For the LoS component, the elevation angle $\theta_{i,1}$ and azimuth angle $\phi_{i,1}$ of user *i* can be expressed as

(7) $$\left\{\begin{array}{cc} & \theta_{i,1}=\arctan\frac{\sqrt{{\left(x-x_{i}\right)}^{2}+{\left(y-y_{i}\right)}^{2}}}{h_{uav}}\\ & \phi_{i,1}=\arctan\frac{y-y_{i}}{x-x_{i}} \end{array}\right.,$$

which are based on the UAV-BS position and user position. For the NLoS components, these angles will be some random values for which the elevation angles $\theta_{i,l}$ generally vary from –90 to 90, while the azimuth angles $\phi_{i,l}$ vary from –180 to 180. In the scenario of blockage, all MPCs will be NLoS components, and the channel H is usually described as a classic Rayleigh fading channel.

The next crucial part in determining the channel response vector $h_{i}$ is its channel gain coefficient $\kappa_{i,l}$. For the LoS component, $\kappa_{i,1}$ mainly depends on the path loss, which is affected by the transmitting distance $D_{i}$ and carrier frequency *f*. The channel gain coefficient for LoS can be described as

(8) $$\begin{array}{c} \kappa_{i,1}=\frac{1}{\left(\frac{4\pi f}{c}\right)\cdot D_{i}^{\alpha }}=\frac{1}{\left(4\pi \lambda \right)\cdot D_{i}^{\alpha }}, \end{array}$$

in which λ is the carrier wavelength, $D_{i}=\sqrt{{\left(x-x_{i}\right)}^{2}+{\left(y-y_{i}\right)}^{2}+h_{uav}^{2}}$ is the linear distance between the UAV-BS and ground user *i*, and α is the LoS path-loss factor. For the NLoS components, we have the following channel gain:

(9) $$\begin{array}{c} \kappa_{i,l}=\frac{\sigma_{f}}{\left(4\pi \lambda \right)\cdot D_{i}^{\beta }}, \end{array}$$

where $\sigma_{f}$ is a small-scale Rayleigh fading factor, and β is the NLoS path-loss factor. Generally, with the existence of LoS, the channel gain will be mainly dominated by the LoS part, and other NLoS parts will sometimes be considered as interferences.

In practice, due to the mobility of the UAV and ground users, some new features, such as non-stationarity and the Doppler shift effect, should also be considered in UAV communications [27,28]. Thus, the time-varying channel response vectors for a URA-equipped UAV-BS should further be expressed as

(10) $$\begin{array}{c} h_{i}\left(t\right)=\sum_{l=1}^{L_{i}}\kappa_{i,l}\left(t\right)a\left(m_{x},m_{y},\theta_{i,l}\left(t\right),\phi_{i,l}\left(t\right)\right)\cdot e^{j\frac{2\pi }{\lambda }\int_{t_{0}}^{t}f_{i,l}\left(t\right)dt}, \end{array}$$

where $f_{i,l}$ is the Doppler frequency. These time-varying factors in Equation (10) are, in essence, mainly determined by the status of the UAV-BS and ground users. As mentioned in Reference [27], only a few mmWave channel sounders have the ability to measure MIMO channels in time-variant environments. MIMO channels will change due to environmental variations, even when transmitters and receivers are static, not to mention in situations, such as a moving UAV-BS serving moving ground users. Due to all these difficulties, which can greatly impact the quality of the channel estimation and the latency of the system, we focus on blind methods to compensate for signal distortion caused by the channel.

## 3. Problem Formulation

In general, in MIMO uplink wireless communications, the basic task of beamforming is to introduce proper weight vectors $w_{i}$ in order to achieve signal recovery against interference. Generally, non-blind beamforming methods will rely on the channel information of H and set the channel response matrix H as prior knowledge that which can be obtained through channel estimation, but this consumes resources. In this paper, the problem that we aim to solve is to derive a blind beamforming scheme at the UAV-BS receivers in order to improve the communication quality of the communication systems. In other words, we aim to calculate the proper beamforming (beam-combining) matrix W for optimal signal recovery at the UAV receivers without detailed information about H. It was shown in Reference [35] that a proper W usually means that the mean squared error (MSE) of the recovered signals can be minimized. Thus, we formulate the blind UAV-BS beamforming problem as a signal recovery MSE minimization problem. We denote by $WX=\hat{S}$ the recovered signals; then, the problem can be expressed as finding a proper W that gives

(11) $$\begin{array}{c} \min\;\;\frac{1}{d\cdot n}\sum_{i=1}^{d}\sum_{j=1}^{n}{\left(|S_{ij}{|}^{2}-|{\hat{S}}_{ij}{|}^{2}\right)}^{2}, \end{array}$$

where *d* is the total user number, and *n* is the number of snapshots. This problem illustrates that we want to minimize the signal recovery MSE for the whole system.

In the noiseless case with perfect CSI, we can directly use W=pinv(H) as a proper beamformer, resulting in

(12) $$\begin{array}{c} WX=\hat{S}=S. \end{array}$$

The recovered signals of the UAV-BS are exactly the original signals sent by ground users. However, in blind situations, we have no clues about what exactly the channel H is. Thus, we try to solve this problem in another way, which is to explore the properties of the UAV’s received signal matrix X.

Generally, some signals in communication systems have a constant modulus (CM) feature, such as signals using BPSK or QPSK modulations. This CM feature can provide useful information for X. We set the user signals with the CM feature as the target signals. For the convenience of illustration, we assume that all the target user signals have unit modulus elements via an appropriate scaler. The classic constant modulus algorithm (CMA) usually regards this signal recovery problem as a constant modulus factorization problem, that is, it makes X=HS or S=WX factorizable under ideal conditions. In essence, the recovered user signals are derived from combinations of vectors in X, and each recovered user signal can be expressed as ${\hat{s_{i}}}^{T}=w_{i}^{T}X$. Consequently, each ${\hat{s}}_{i}^{T}$ can be seen as a projection onto the row span of X. As we now have the signal CM property, for a more distinct illustration, the problem mentioned in Equation (11) can be transformed into

(13) $$\begin{array}{c} \begin{array}{cc} \; & \min\;\;\frac{1}{d\cdot n}\sum_{i=1}^{d}\sum_{j=1}^{n}{\left(\;1\;-|{\hat{S}}_{ij}{|}^{2}\right)}^{2}\\ \; & s.t.\;{\hat{s}}_{i}^{T}={\left(\hat{S}\right)}_{i}\in \mathrm{rowspan}\left(X\right),\;\forall i\in \left\{1,\ldots ,d\right\}. \end{array} \end{array}$$

This equation guarantees that the derived beamformer explores the CM feature in order to achieve optimal beamforming for signal recovery accuracy, and simultaneously makes sure that the recovered signals belong to specific UAV-BS receivers. We will introduce an analytical blind method called the analytical constant modulus algorithm (ACMA) in order to solve this problem in the following sections.

## 4. Solution of the Problem

We first assume that the ground users are transmitting independent signals with enough phase richness, which means that S is a full rank and the CM factorization can be unique. In practice, some trivial transformations, such as phase invariance, will sometimes cause admissible non-uniqueness, but the uniqueness of recovered signals $\hat{S}$ can be guaranteed for n→∞. Once the uniqueness is guaranteed, we can make sure that the recovered user signals at the UAV-BS site are the target signals originally sent by ground users [32]. To achieve a minimal signal recovery MSE for the whole system, we can pay attention to each user signal. Ideally, each recovered user signal should have unit modulus elements, as we assumed before, that satisfy

(14a) $$\begin{array}{cc} \; & {\hat{s}}_{i}^{T}=\left[1,\cdots ,1\right],\;\forall i\in \left\{1,\ldots ,d\right\} \end{array}$$

(14b) $$\begin{array}{cc} \; & s.t.\;{\hat{s}}_{i}^{T}={\left(\hat{S}\right)}_{i}\in \mathrm{rowspan}\left(X\right),\;\forall i\in \left\{1,\ldots ,d\right\}. \end{array}$$

As mentioned in the previous sections, the UAV-BS’s received signal matrix X is the only data that we can acquire in the system. For an antenna-given $X\in C^{m\times n}$, the first step in the ACMA scheme is to separate the signal space from the interference space. To divide spaces in X, we need to apply singular value decomposition (SVD) to X.

Let $\mathrm{SVD}\left(X\right)=U\Sigma V^{*}$, where $U\in C^{m\times m}$, $\Sigma \in R^{m\times n}$, and $V\in C^{n\times n}$. By estimating the rank of X, we can first obtain the number of received signals. One direct way is to check how many singular values are non-zero in Σ; the number of non-zero values is equal to the number of transmitted signals. Remember that ground users are transmitting *d* independent signals, and the received signal matrix X is, in essence, the combination of transmitted signals $s_{i}$ through channels, so there are actually *d* independent row vectors in the row space of X. The SVD operation gives us the singular non-zero values and their corresponding vectors, which can actually form the basis of rowspan(X). This SVD of X first gives us the number of transmitted signals; then, we extract the signal space from X, which is helpful for the following operations. Notice that the antenna array will sometimes receive all of the signals that appear in the system, including non-CM (non-user) ones, so the rank of X is not always precisely *d* in the presence of non-CM signals. We use δ as the total signal number; then, we have δ non-zero values in Σ. In fact, the SVD of X can only extract the signal space, not the target CM signal space. To obtain the CM signal space, more operations are needed.

Let $\hat{V}\in C^{\delta \times n}$ be the submatrix of V that contains the singular vectors corresponding to δ singular non-zero values; then, we have that the rows of $\hat{V}$ are actually the orthogonal basis of rowspan(X), which can be seen as the signal space. Thus, we have

(15) $$\begin{array}{c} s_{i}^{T}\in \mathrm{rowspan}\left(X\right)\Leftrightarrow s_{i}^{T}=\omega_{i}^{T}\hat{V},\;\forall i\in \left\{1,\;\ldots ,\;\delta \right\}, \end{array}$$

where the weight vectors $w_{i}$ are acting on the signal space. Notice that, here, $s_{i}^{T},\;\forall i\in \left\{1,\ldots ,\delta \right\}$ are not yet the recovered target user signals, and some non-CM signals are still in the subspace. In other words, the SVD operation of X extracts the *d* user signals together with δ−d interference signals. Clearly, this cannot yet meet the requirements for user signal recovery. In the following, we will separate user signals from interference signals by using the CM property.

We know that $\hat{V}=\left[v_{1},\ldots ,v_{n}\right]$, where $v_{j},\forall j\in \left\{1,\ldots ,n\right\}$ is the j−th column of $\hat{V}$; then, we can rewrite Equation (14a) as

(16) $$\begin{array}{c} \begin{array}{cc} {\hat{s}}_{i}^{T} & =\left[1,\ldots ,1\right],\forall i\in \left\{1,\ldots ,d\right\}\\ \; & \Leftrightarrow \left[{\left|{\left({\hat{s}}_{i}^{T}\right)}_{1}\right|}^{2}\ldots {\left|{\left({\hat{s}}_{i}^{T}\right)}_{n}\right|}^{2}\right]=\left[1,\ldots ,1\right],\;\forall i\in \left\{1,\ldots ,d\right\}\\ \; & \Leftrightarrow w_{i}^{T}v_{j}v_{j}^{H}w_{i}^{*}=1,\;\forall i\in \left\{1,\ldots ,d\right\},\forall j\in \left\{1,\ldots ,n\right\}. \end{array} \end{array}$$

We first define $P_{j}=v_{j}v_{j}^{H}\in C^{\delta \times \delta },\forall j\in \left\{1,\ldots ,n\right\}$; then, the CM property in Equation (16) can be expressed as

(17) $${\hat{s}}_{i}=\left[\begin{array}{c} w_{i}^{T}P_{1}w_{i}^{*}\\ \vdots \\ w_{i}^{T}P_{j}w_{i}^{*}\\ \vdots \\ w_{i}^{T}P_{n}w_{i}^{*} \end{array}\right]=\left[\begin{array}{c} 1\\ \vdots \\ 1 \end{array}\right].$$

Now, in the extracted signal space, our goal is to find linearly independent beamformers $w_{i}$ for each user that can enforce this signal CM property. Once we have found all of the $w_{i}$, we will have separated the user signal space (the target CM signal space) from the non-user signal space, and the blind beamforming problem for the UAV-BS can be solved.

For a better illustration, we can transform Equation (17) into a matrix computation form. In the following, we will temporarily drop the index *i* of w for clarity, i.e., w stands for all solutions $w_{i},\forall i\in \left\{1,\ldots ,d\right\}$. We define two operators, vec(·) and mat(·), where vec(·) will stack all elements of a matrix into a vector by column, and mat(·) is the inverse operator of vec(·).

Let $p_{j}=vec\left(P_{j}\right)\in C^{\delta^{2}}$; with the structure of $y=vec\left(w^{*}w^{T}\right)=w⊗w^{*}\in C^{\delta^{2}}$, we have the original CM elements in which $w^{T}P_{j}w^{*}=1$ is equal to $p_{j}^{T}y=1$; then, Equation (17) can be expressed as

(18) $$Py=\left[\begin{array}{c} 1\\ \vdots \\ 1 \end{array}\right],where\;P=\left[\begin{array}{c} p_{1}^{T}\\ \vdots \\ p_{j}^{T}\\ \vdots \\ p_{n}^{T} \end{array}\right],y=w⊗w^{*}.$$

For greater convenience of computation, we can try to use some linear algebraic methods to transform Equation (18) into a more manageable form. One reasonable choice can be Householder transformation (HT) [32,36], though other options that can achieve the same goal are also acceptable. Let

(19) $$Q=I-2\frac{qq^{*}}{q^{*}q},\;q=\left[\begin{array}{c} 1\\ \vdots \\ 1 \end{array}\right]-\left[\begin{array}{c} \sqrt{n}\\ 0\\ \vdots \\ 0 \end{array}\right].$$

Then, by applying this Q to P, we have separated the system into two parts:

(20) $$QPy=\left[\begin{array}{c} {\hat{p}}_{1}\\ \hat{P} \end{array}\right]y\Leftrightarrow \left\{\begin{array}{cc} & \left(1\right)\;{\hat{p}}_{1}y=\sqrt{n}\\ & \left(2\right)\;\hat{P}y=0 \end{array}\right.\;,\;\left.\begin{array}{cc} \; & {\hat{p}}_{1}:1\times \delta^{2}\\ \; & \hat{P}:\left(n-1\right)\times \delta^{2} \end{array}\right.\;.$$

After this transformation, we can directly focus on the second part of this system, which is easier to solve than the system Py=1. The first part corresponds to the scaling of the vectors, which will be addressed later. Now, we try to find a solution y that satisfies

(21) $$\begin{array}{c} \hat{P}y=\left[\begin{array}{c} 0\\ \vdots \\ 0 \end{array}\right],y=w⊗w^{*}. \end{array}$$

Due to the existence of multiple ground users, there will be more than one solution to the system. We require the number of snapshots *n* to be larger than $\delta^{2}$; otherwise, there will be no solutions to the system. One simple and direct way to calculate these solutions is to employ SVD for $\hat{P}$ because the solutions that make $\hat{P}y=0$ can be seen as the orthogonal basis of the null-space ($\hat{P}$). Thus, the singular vectors corresponding to singular zero values can be seen as the solutions. In the case of noisy channels, we retrieve the singular vectors corresponding to the smallest singular values of $\hat{P}$. This operation can correctly estimate the number of user signals in the signal space.

However, as we mentioned before, each CM solution y corresponding to each ground user in the UAV-BS system should have the specific structure $w⊗w^{*}$. In common wireless UAV-BS communications, the sample size is generally much larger than the number of users served, which leads to an overdetermined system. We require $n>\delta^{2}$; hence, the set of independent solutions y is not unique. Thus, we need the condition $y=w⊗w^{*}$ to restrict the solution space. Simple SVD operations cannot guarantee this structure; it is not appropriate to compute the solutions y and hope that they have the specified structure [32]. Operations are needed to make sure that each solution can be expressed as $w⊗w^{*}$. We will simply introduce the extended QZ iteration method in the ACMA to solve this structural problem.

In general, the CM solution space that satisfies Equation (21) can be written as

(22) $$\begin{array}{c} y=c_{1}\beta_{1}+c_{2}\beta_{2}+\ldots +c_{\hat{d}}\beta_{\hat{d}}=w⊗w^{*}, \end{array}$$

where $\left\{c_{1},\ldots ,c_{\hat{d}}\right\}$ is a set of constants, $\left\{\beta_{1},\ldots ,\beta_{\hat{d}}\right\}$ is the basis of the kernel of $\hat{P}$, and $\hat{d}$ is the dimension of *Ker* $\left(\hat{P}\right)$. The linearly independent y corresponds to linearly independent w, which now leads to a linearly independent set of constants. Let $\mathrm{SVD}\left(\hat{P}\right)=U_{p}\Sigma_{p}{V_{p}}^{*}$, as mentioned above, be the basis of *Ker* $\left(\hat{P}\right)$, which can be estimated as the singular vectors corresponding to singular zero values. The dimension of *Ker* $\left(\hat{P}\right)$ is equal to the number of singular zero values, which is also the number of target user signals, so we have $\hat{d}=d$ (proved in Reference [32]). Then, the last *d* rows of ${V_{p}}^{*}$ corresponding to singular zero values can form a basis $\left\{\beta_{1},\ldots ,\beta_{d}\right\}$. After finding the basis of this CM solution space, once we obtain $\left\{c_{1},\ldots ,c_{d}\right\}$, we will have separated the CM signal space from the non-CM ones. Notice that, here, we say “singular zero values” because this is under the ideal conditions, which means that there is no noise in the system. When additive noise is applied to the system, there will be a threshold for deciding when small singular values can be seen as “singular zero values”. The discussion about the threshold will be introduced in the next section.

Let $B_{k}=mat\left(\beta_{k}\right),k\in \left\{1,\ldots ,d\right\}$, and Y=mat(y); then, Equation (22) can be expressed as

(23) $$\begin{array}{c} Y=c_{1}B_{1}+\ldots +c_{d}B_{d}=w^{*}\cdot w^{T}. \end{array}$$

A proper set of constants can make the result of Equation (23) rank 1 and positive semidefinite; hence, it can be factorized as $w^{*}\cdot w^{T}$. Assume that we already have the weight vectors $w_{1}\ldots w_{d}$; then, $\beta_{i}=w_{i}⊗{w_{i}}^{*},i\in \left\{1,\ldots ,d\right\}$ can form the basis of $Ker(\hat{P})$, and each $Y_{i}$ can be factorized as

(24) $$\begin{array}{c} Y_{i}=\lambda_{i1}{w_{1}}^{*}w_{1}^{T}+\lambda_{i2}{w_{2}}^{*}w_{2}^{T}+\ldots +\lambda_{id}{w_{d}}^{*}w_{d}^{T}=W^{*}\Lambda_{i}W^{T}. \end{array}$$

If we could find matrix Q and Z to make $QY_{i}Z=\Lambda_{i}$, then the set of constants $\left\{c_{1},\ldots ,c_{d}\right\}$ corresponding to each user signal (making the solution rank 1) could be derived from the eigenvalue matrices $\Lambda_{i}$. Consequently, the UAV-BS beamforming problem can be seen as an eigenvalue problem. To reduce the calculation complexity and save the calculation cost in the UAV-BS, it is not necessary to fully diagonalize $Y_{i}$, as eigenvalues can also be obtained if $QY_{i}Z$ are upper-triangular matrices.

The extended QZ iteration sets the initial $Q^{\left(0\right)}=Z^{\left(0\right)}=I$. For the k−th iteration, this method aims to make

(25) $$\begin{array}{c} \begin{array}{cc} \; & min\;||Q^{\left(k\right)}\left(Y_{1}Z^{\left(k-1\right)}\right){\left|\right|}_{LF}^{2}+\ldots +\left|\right|Q^{\left(k\right)}\left(Y_{d}Z^{\left(k-1\right)}\right){\left|\right|}_{LF}^{2}\\ \; & min\;||\left(Q^{\left(k\right)}Y_{1}\right)Z^{\left(k\right)}{\left|\right|}_{LF}^{2}+\ldots +\left|\right|\left(Q^{\left(k\right)}Y_{d}\right)Z^{\left(k\right)}{\left|\right|}_{LF}^{2} \end{array}, \end{array}$$

which necessitates making the lower-triangular part the minimal norm. Methods, such as HT and SVD, can be used to construct upper-triangular $R_{i}=QY_{i}Z$ [32]. With the derived $R_{i}$, we extract the diagonal entries of each $R_{i}$ and form a coefficient matrix:

(26) $$\begin{array}{c} C={\left[\begin{array}{ccc} (R_{1}{)}_{11} & \ldots & (R_{1}{)}_{dd}\\ \vdots & \; & \vdots \\ (R_{d}{)}_{11} & \ldots & (R_{d}{)}_{dd} \end{array}\right]}^{-1}. \end{array}$$

Finally, each set of constants $\left\{c_{1},\cdots ,c_{d}\right\}$ is given by rows of C. Hence, each $Y_{i}=w^{*}\cdot w^{T}$ can be calculated, and the beamformers $w_{i}$ for target user signal recovery $s_{i}^{T}=w_{i}^{T}\hat{V}$ can be estimated as the singular vectors corresponding to the largest singular value in each $Y_{i}$. The user signal space can be successfully separated from the interference space. However, sometimes, we still need to run the Gerchberg–Saxton algorithm (GSA) several times to solve the phase invariance problem in order to make the solutions near optimal. At the k−th iteration, the update rule is

(27) $$\begin{array}{c} w_{i}^{T}\left(k+1\right)=\left[\begin{array}{c} \frac{{\left(w_{i}^{T}\left(k\right)\hat{V}\right)}_{1}}{|{\left(w_{i}^{T}\left(k\right)\hat{V}\right)}_{1}|},\ldots ,\frac{{\left(w_{i}^{T}\left(k\right)\hat{V}\right)}_{n}}{|{\left(w_{i}^{T}\left(k\right)\hat{V}\right)}_{n}|} \end{array}\right]. \end{array}$$

## 5. Performance Evaluation

In this section, we perform numerical simulations to test the efficiency of the analytical method for blind beamforming in a UAV-BS. We consider an uplink wireless communication scenario in which a UAV-BS serves *d* ground users in a specific urban region—for instance, a 200 m × 200 m square region. In this region, the UAV-BS hovers in the sky, while the users stay on the ground. The time-varying channel is considered according to the model in Equation (10). We assume that the UAV moves at a speed of 10 m/s [28], while the ground users maintain static. The carrier frequency is set to 28 GHz in the mmWave band. So, $f_{Doppler}$ can be easily derived. The positions of the UAV-BS and ground users are randomly distributed in the horizontal direction. For each position setting in each simulation run, we average the simulation results over 100 times.

The UAV-BS is equipped with an $m=m_{x}\times m_{y}$ element URA, while each ground user is equipped with a single antenna. In this uplink process, ground users transmit independent and randomly generated signals by using QPSK modulations with *n* snapshots. Each signal is assumed to have $L_{i}=4$ MPCs with the existence of LoS and the three strongest NLoS components; thus, the channel in this scenario can be regarded as a mixture of LoS and NLoS parts. We set the path-loss factors α=0.95 and β=2.25 in Equations (8) and (9) according to the mmWave channel measurements in Reference [37]. The small-scale factor $\sigma_{f}$ can be set to a small value, such as 0.01. By combining the channel model mentioned in Section 2.2 and the parameters in this section, we can simulate different channels for the tests of this method. According to detailed parameter and environment settings in this section, combined with the algorithm and models mentioned above, we can have the antenna outputs matrix X, signal matrix s and the beamforming matrix H. Then, we can easily evaluate the algorithm performance in this specific-simulated application scenario.

**Figure 2:**
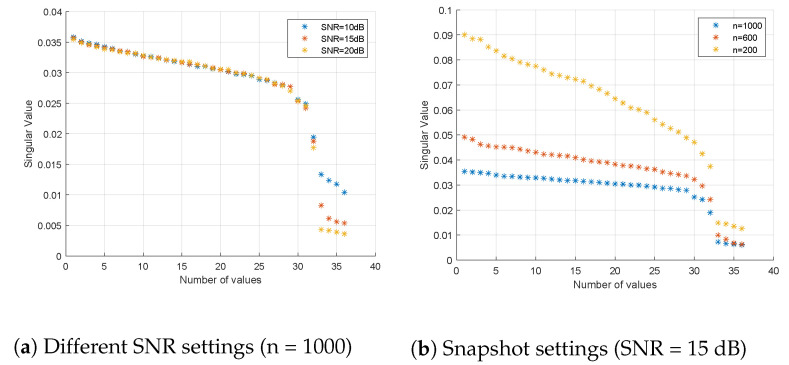
Estimation of the number of ground users by examining small singular values when using different SNR/snapshot settings.

In the following simulations, additive Gaussian white noise is added to the system. We first assume that m=4×4, and there are δ=6 signals that appear in the system; four of them are transmitted by ground users (target CM signals). By exploring the number of singular near-zero values after *SVD* $\left(\hat{P}\right)$, we can estimate how many ground users are located in the region served by the UAV-BS. As shown in Figure 2, we tested the efficiency of the estimation of the number of ground users under different SNR and snapshot settings.

Due to the existence of Gaussian white noise, the singular values corresponding to the ground users are raised above zero. However, there is still a gap between the near-zero values and other values. With the increasing SNR and number of snapshots, the gap becomes more distinct. A reasonable threshold was given in Reference [32], which the large singular values should satisfy (with a probability better than 95%)

(28) $$\begin{array}{c} min\left(large\;singular\;value\right)>\frac{1}{\sqrt{n}}-\frac{\delta -0.5}{n}. \end{array}$$

We then tried to evaluate the performance of the analytical method in minimizing the system’s MSE under different channel settings. For the LoS channel, the LoS component dominates the channel response. For the NLoS channel, all MPCs are regarded as NLoS components. The Rayleigh fading channel represents an ideal NLoS situation in which H∼Rayleigh(1). We used the classic MMSE method, random beamforming, and the Artificial Bee Colony (ABC) method to compare with the analytical method. As we do not know the channel H in a blind situation, the MMSE receiver cannot be directly employed in practice. However, here, we assume that the MMSE receiver already knows the channel. The classic MMSE will give us a weight vector

(29) $$\begin{array}{c} W_{MMSE}=H^{H}{\left(HH^{H}+\frac{\sigma^{2}}{P}I\right)}^{-1}, \end{array}$$

where $\sigma^{2}$ is the system noise power, and *P* is the system signal power; $\frac{\sigma^{2}}{P}$ is actually $SNR^{-1}$ on the natural scale. The random beamformer is defined as $W_{rand}=e^{j\Psi }$, where each element of the random phase matrix Ψ will give us a random phase within [0,2π] [20]. The ABC method here is slightly different from the ABC beamforming process in Reference [20]; we transform it into an uplink process here. In this test, we set the food source number Ns=200 with iteration=500.

**Figure 3:**
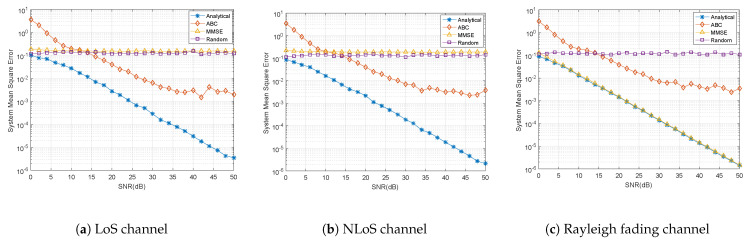
The system’s MSE using different beamforming methods with different channels.

From Figure 3, we can observe that, for the LoS and NLoS channel settings, the analytical blind beamforming achieves better performance in minimizing the system’s MSE, while the MMSE and ABC receivers do not perform as well. For the Rayleigh fading channel, the efficiency of the analytical method is close to that of the MMSE because, in this situation, noise is the dominant force affecting the signal recovery. The actual values depend on the randomness of the noise and the channel distortion. Nevertheless, the analytical blind beamforming is useful in most common scenarios.

**Figure 4:**
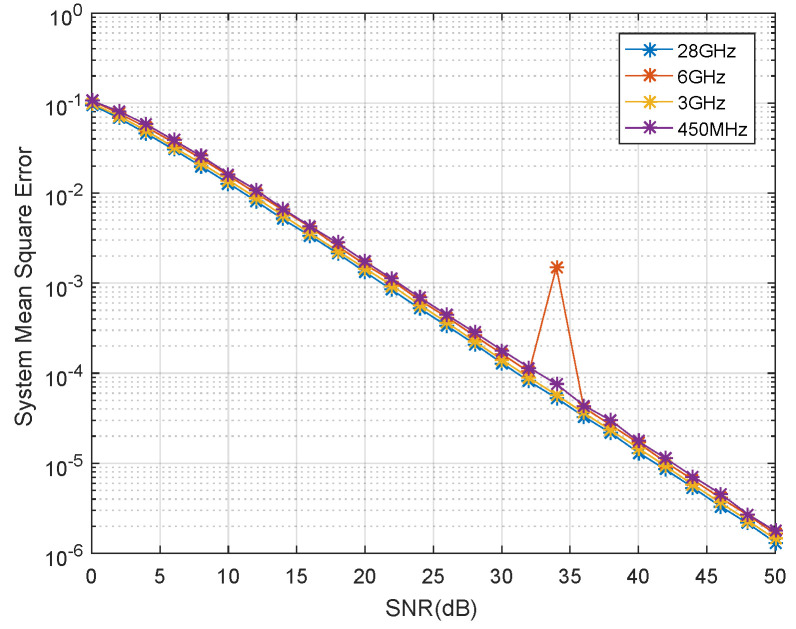
The MSE performance of the analytical method with different frequency bands.

We also tested the ACMA beamforming method under some sub-6-GHz bands. As shown in Figure 4, the results are very close to each other. The carrier frequency in our simulations mainly decides the values of the path loss and $f_{Doppler}$, but it does not affect the performance of the method in achieving a minimal system MSE.

**Figure 5:**
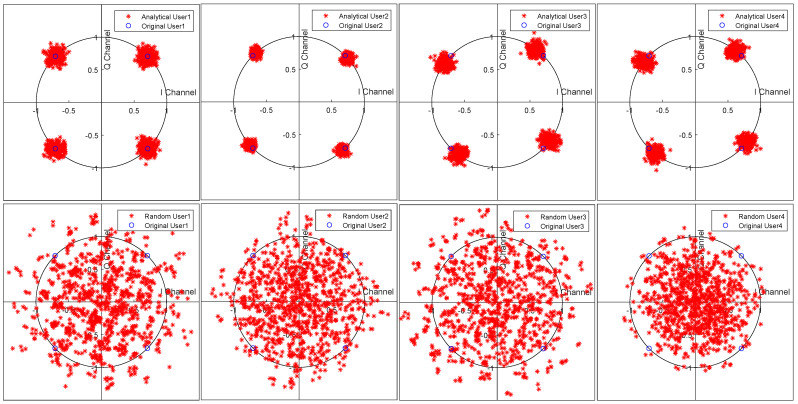
Constellation diagrams of the received user signals when SNR =15dB. The upper row shows the recovered signals of four different users using the ACMA with the original constellation. The bottom row shows the signals when using random beamforming.

Figure 5 shows the constellations of the originally sent user signals and recovered user signals. It directly shows the performance of the ACMA in beamforming for signal recovery. Compared with random beamforming, the analytical method can successfully derive both the amplitude and phase information. Due to the phase ambiguity of blind beamforming methods, sometimes, there are phase differences between the original signals and the recovered ones. Operations, such as differential encoding, can be applied to cancel the phase differences. However, we mainly focused on blind beamforming for signal recovery; thus, we have omitted the details about the encoding part.

**Figure 6:**
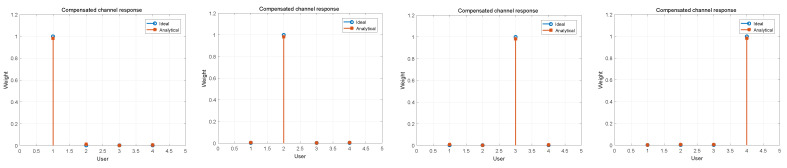
Compensated channel response of four different users with the ACMA in comparison with ideal beamforming when SNR =10dB.

In Figure 6, we show the compensated channel response for d=4 different users after applying analytical beamforming. We can see that the compensated channel wH looks the same as an impulse. This, in essence, shows that the channel effects on each transmitted user signal are nearly erased at the UAV-BS’s receiver. In other words, the ACMA receivers only recover user signals ($w_{i}Hs=s_{i}$), which can be seen as directing the receiving antennas towards different users.

Then, we evaluate the system’s sum rate when applying the analytical method under different settings. The system’s sum rate can be described as

(30) $$\begin{array}{c} Sumrate=\sum_{i=1}^{d}\log_{2}\left(1+\frac{P\cdot |h_{i}^{H}w_{i}{|}^{2}}{\sigma^{2}}\right), \end{array}$$

which summarizes achievable rates of *d* ground users. Here, the signal power *P* and noise power $\sigma^{2}$ are still calculated on the natural scale. For better illustration, we transform them into dB in the figures. We introduced approximate beamforming as an upper bound in our tests. Approximate beamforming is regarded as an ideal beamforming in which the beam gains are zeros along the non-user directions and are significant along the user directions [20].

**Figure 7:**
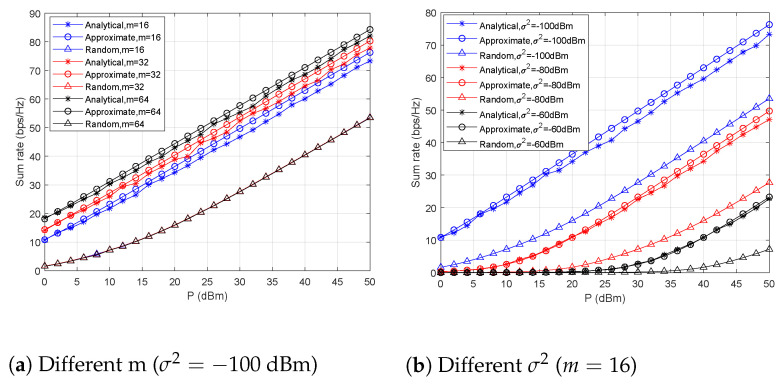
The system’s sum rate performance after analytical blind beamforming.

In this experiment, we first evaluated the performance of these three beamforming methods with a settled noise power of $\sigma^{2}=-100$ dBm and varying antenna numbers *m*. Then, we used a 16-element URA to evaluate the system’s sum rate for varying $\sigma^{2}$. As shown in Figure 7, under different *m* and $\sigma^{2}$ settings, the system sum rates achieved with the analytical method are close to the approximate ones; moreover, they are much better than with the random values.

**Figure 8:**
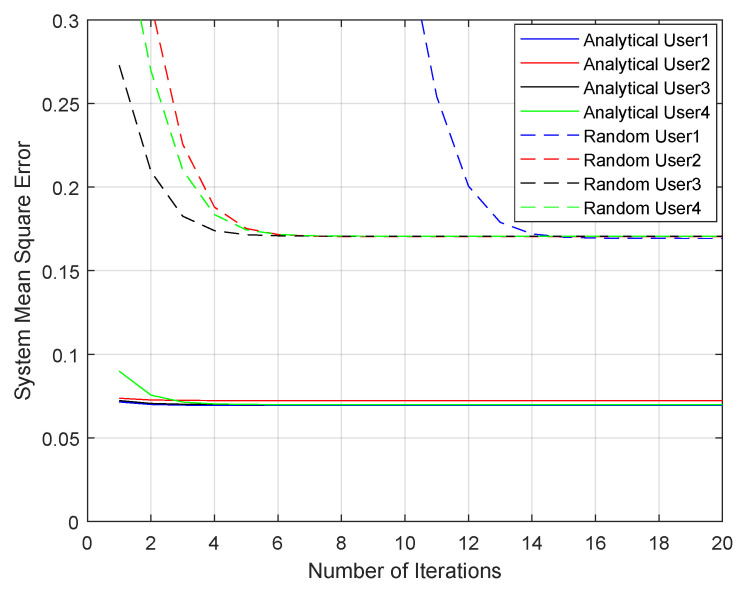
GSA convergence speed of four users using random initial points or points calculated with the ACMA when SNR = 20 dB.

As mentioned in Section 4, the Gerchberg–Saxton algorithm (GSA) is used for the phase-retrieval problem. We proceeded with some iterations of the GSA for both analytically and randomly calculated weight vectors $w_{i}$ for more accuracy. The convergence speed of GSA was tested in our simulations. As shown in Figure 8, the beamformers $w_{i}$ obtained with the analytical method can achieve a very fast convergence speed, while the ones derived from random beamforming surely need more iterations to guarantee that they converge to their optimal solutions.

After testing the performance of the analytical method in terms of the signal recovery accuracy and sum rate, we then tested its performance in terms of complexity. In Table 1, we compare it with classic non-blind methods (LS channel estimation/MMSE estimation + MMSE receiver) and the ABC method. As different methods depend on different parameters, sometimes, it is hard to directly compare the complexity in one united form. Thus, we compare these methods when they achieve comparable MSE results and introduce an equivalent complexity. In our settings, we need at least m⩾d and $n⩾d^{2}+1$, so we can transform O(dnm) into $O(d^{4})$ [32]. When d=4, we need about $L_{GSA}=20$, Ns=100, and $L_{ABC}=300$ to make the CMA and ABC converge to their optimal results, so we obtain $O\left(L_{GSA}\right)\approx O\left(d^{2}\right)$, $O\left(Ns\right)\approx O\left(d^{3}\right)$, and $O\left(L_{ABC}\right)\approx O\left(d^{4}\right)$. The equivalent complexity of each method will depend on the actual parameter settings.

**Figure 9:**
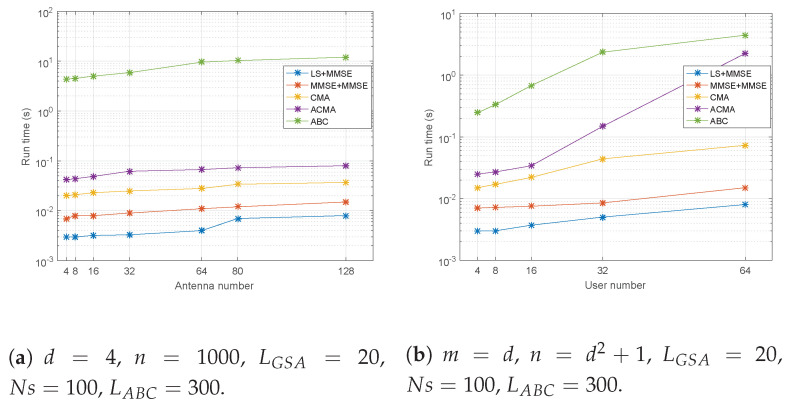
Simulation runtimes for different parameter settings.

We then tested the simulation runtimes under specific parameter settings, as shown in Figure 9. We can see that the ACMA can achieve a comparable runtime with that of the classic methods with respect to different values of *m*. However, with an increasing user number, there is a dramatic runtime increase for the ACMA, but it is still more efficient than ABC. In fact, when the user number increases, ABC needs more food sources and iterations to obtain good results, so we set $N_{s}=100$ and $L_{ABC}=300$ for only the controlled variables.

In relation to Figure 3, there is a trade-off problem between the complexity and the system MSE. Indeed, the analytical method is more complex than the traditional MMSE method, but it achieves more accurate signal recovery beamforming when considering new features in the UAV communication channels. Accurate channel estimation is crucial for non-blind methods, but it is still difficult to acquire due to the time variance and other features [27]. From another perspective, battery endurance and energy cost are two inevitable factors in UAV-assisted networks that must still be solved, though non-blind methods usually need pilots or training sequences, which impact the bandwidth efficiency, power consumption, and battery life [38]. The computational complexity is not as difficult to deal with because chips are becoming more capable.

Through the evaluations in this section, we can observe that, when applied to a simulated UAV-BS system, the analytical blind beamforming method can achieve good MSE performance in terms of signal recovery and a reasonable system sum rate. Complex and difficult MIMO channel estimation is omitted to save costs. In other words, this method can successfully solve the MIMO UAV-BS beamforming problem without CSI. At the same time, it can achieve a reasonable computational complexity when there are not too many ground users. One possible UAV-BS application scenario can be a situation in which some specific users/target signals need higher-quality communications.

## 6. Conclusions

In this paper, we investigated the blind beamforming method with the ACMA at URA receivers for an MIMO UAV-BS system in mmWave bands. We firstly formulated the UAV-BS beamforming problem as a system MSE minimization problem. Then, we introduced the analytical method, ACMA, in order to derive a proper beamformer for signal recovery. Instead of relying on detailed CSI, the analytical method only explores information from antenna outputs. This method separates the user signal space from other interference spaces by using algebraic operations, such as SVD and diagonalization; then, it uses a specific structure to restrict the signal space to obtain a proper beamformer. We evaluated this method under different settings, such as different channels, different antenna settings, and different noise environments. New channel features, such as UAV mobility and time variance, were taken into consideration. The simulation results show that the analytical method exhibits good performance in terms of minimizing the system MSE, and, at the same time, it achieves a reasonable system sum rate. When the UAV-BS is not serving too many users, it achieves a complexity that is comparable with that of the MMSE method. Future research might study the overall power consumption increased by computation and transmission length. Practical experiments might be included in future work.

## Figures and Tables

**Table 1:** Comparison of the complexity of the methods when achieving comparable system MSE values (notations: *m*: antenna number, *d*: user number, *n*: snapshots, Ns: food source number, $L_{GSA}$: iteration number of the GSA, $L_{ABC}$: iteration number of ABC).

Method	Complex Operations	Equivalent Complexity
LS+MMSE	$m^{2}d+2d^{2}m+dmn$	$O(d^{4})$
MMSE+MMSE	$5m^{2}d+2d^{2}m+dmn$	$O(d^{4})$
CMA	$L_{GSA}2dmn+2m^{2}n$	$O(d^{6})$
ACMA	$9{\left(d^{2}\right)}^{2}n+9m^{2}n$	$O(d^{6})$
ABC	$N_{s}dm+L_{ABC}\left(2N_{s}+1\right)dm$	$O(d^{7})$

## Data Availability

Data are only available upon request due to restrictions regarding, e.g., privacy and ethics. The data presented in this study are available from the corresponding author upon request. The data are not publicly available due to their relation to other ongoing research.
